# Leptin, Adiponectin, and Sam68 in Bone Metastasis from Breast Cancer

**DOI:** 10.3390/ijms21031051

**Published:** 2020-02-05

**Authors:** Paola Maroni

**Affiliations:** IRCCS Istituto Ortopedico Galeazzi, Via R. Galeazzi 4, 20161 Milano, Italy; paola.maroni@grupposandonato.it; Tel.: +39-02-66214759

**Keywords:** bone metastasis, bone marrow adipose tissue (BMAT), adipokines, Src-associated in mitosis of 68 kDa (Sam68)

## Abstract

The most serious aspect of neoplastic disease is the spread of cancer cells to secondary sites. Skeletal metastases can escape detection long after treatment of the primary tumour and follow-up. Bone tissue is a breeding ground for many types of cancer cells, especially those derived from the breast, prostate, and lung. Despite advances in diagnosis and therapeutic strategies, bone metastases still have a profound impact on quality of life and survival and are often responsible for the fatal outcome of the disease. Bone and the bone marrow environment contain a wide variety of cells. No longer considered a passive filler, bone marrow adipocytes have emerged as critical contributors to cancer progression. Released by adipocytes, adipokines are soluble factors with hormone-like functions and are currently believed to affect tumour development. Src-associated in mitosis of 68 kDa (Sam68), originally discovered as a protein physically associated with and phosphorylated by c-Src during mitosis, is now recognised as an important RNA-binding protein linked to tumour onset and progression of disease. Sam68 also regulates splicing events and recent evidence reports that dysregulation of these events is a key step in neoplastic transformation and tumour progression. The present review reports recent findings on adipokines and Sam68 and their role in breast cancer progression and metastasis.

## 1. Introduction

Bone metastases are a leading cause of death in patients with advanced breast and prostate cancers. Mineral content, matrix composition, extreme rigidity, highly hypoxic environment, acidic pH, and a high concentration of extracellular calcium all make the bone an attractive site for colonization by cancer cells. Furthermore, residing within the bone matrix is a vast array of cytokines, growth factors, and matrix proteins that can contribute to the development of bone metastases. Host cells in the tumour microenvironment drive metastatic behaviour and influence tumour growth and progression. In addition, the bone marrow environment contains a variety of cells, including mesenchymal stem cells (MSC), which give rise to several cell lineages such as osteoblasts and adipocytes.

Once considered passive fillers of the bone marrow niche or cells that occupy the space after trabecular bone loss, adipocytes are now known to be endocrine targets and to possess endocrine-like functions: besides responding to growth hormones, insulin, and thyroid hormone, they release a plethora of adipokines among which leptin and adiponectin [1]. The present review focuses on these two factors and their possible roles in bone marrow colonization by breast carcinoma cells. Furthermore, I discuss the Src-associated in mitosis of 68 kDa (Sam68), a signal transduction and activation of RNA (STAR) protein and eclectic component of signal transduction of leptin, which is strongly involved in breast cancer progression. The clinical consequences of bone metastases from breast cancer, as for other types of cancer, are often associated with reduced quality and life expectancy of patients due to skeletal-related events (SRE), which include pathological bone fractures, spinal cord compression, severe pain, and hypercalcaemia [2,3,4], with an increase in healthcare costs. Shedding light on the mechanisms that link adipokines and breast cancer progression and metastasis could help to identify new therapeutic opportunities.

## 2. Bone Marrow Adipose Tissue (BMAT), the Third Fat Depot

Now recognized as the body’s largest endocrine organ, adipose tissue secretes a variety of different factors: adipokines, cytokines, and exosomal microRNAs [5]. The two main subtypes of adipose tissue are white and brown adipose tissue (WAT and BAT, respectively). WAT, the more abundant of the two, is localized subcutaneously and viscerally. Its main function is to release energy from lipid stores as needed. In contrast, BAT is found in variable amounts in more distinct areas in adult humans. BAT works in adaptive thermogenesis through the uncoupling of ATP production and substrate oxidation [6].

A less known third type of adipose tissue is BMAT, which has garnered recent interest owing to its probable role in cancer progression. This discovery is particularly relevant for breast and prostate carcinoma-derived metastasis and for multiple myeloma arising in the bone. BMAT constitutes a major component of the bone marrow microenvironment in which the number of adipocytes increases with age: 40–70% of appendicular skeleton of lean adults (after age 25 years) is occupied by adipocytes [7,8], with continued gradual accumulation of BMAT throughout life [9].

**Figure 1:**
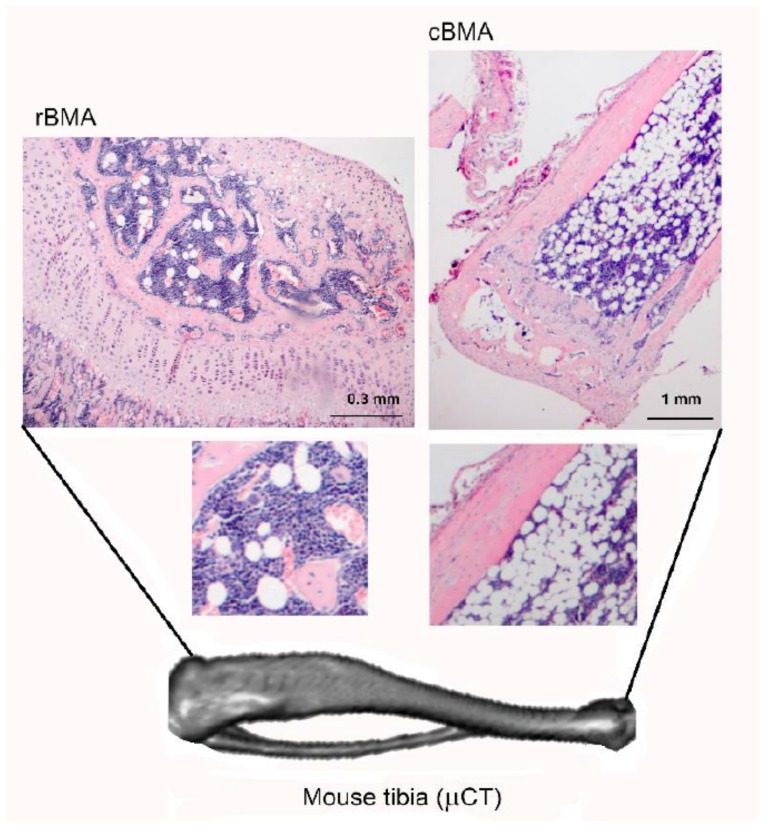
Distribution of regulated (rBMA) and constitutive (cBMA) bone marrow adipocytes in the mouse tibia. Original figure courtesy of the author.

Two types of adipocytes make up BMAT: constitutive bone marrow adipocytes (cBMA) residing in distal areas of bones and regulated bone marrow adipocytes (rBMA) found in more metabolically active bone sites (Figure 1). 

The two adipocyte populations differ also in lipid content: rBMA stores prevalently saturated fats whereas cBMA stores unsaturated fats [10]. rBMA undergo constant changes during aging and promptly react to changes in nutritional, hormonal, and temperature states.

Furthermore, BMAT serves as an energy reservoir and a producer of cytokines and adipokines. Its unique characteristics make it different from WAT or BAT. BMAs are derived from multipotent mesenchymal progenitors that express Osterix, a transcription factor essential for osteoblastogenesis. In further contrast to WAT, BMAT expands under conditions of energy restriction and does not seem to be affected by energy surplus, at least not in humans. BMAs can influence the balance between adipogenesis and osteoblastogenesis: through the secretion of extracellular vesicles containing adipogenic mRNA transcript, BMAs weaken osteoblast lineage commitment [11]. BMAs can thus act as negative regulators of osteoblastogenesis by reducing the number of osteoblasts and decrease bone formation and resistance as a consequence. BMAs also inhibit haematopoiesis. Indeed, the haematopoietic activity in bone marrow is inversely correlated with adiposity [12]. The influence of BMAs in the context of cancer development and progression is still poorly understood, however.

**Figure 2:**
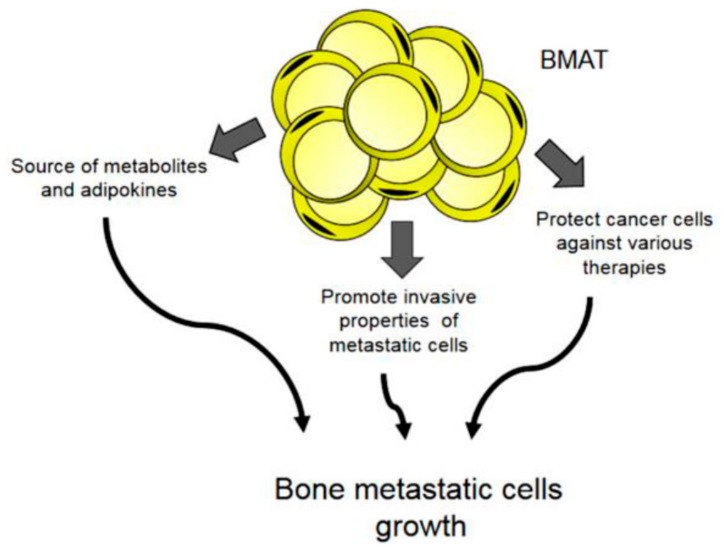
Potential contribution of BMAT to bone metastatic growth.

The contribution of BMAs to tumour growth and metastasis has been amply discussed (Figure 2). Adipocytes exert a critical role in disease progression in the tumour microenvironment by providing cancer cells with fatty acids, pro-inflammatory cytokines, and proteases [13,14,15]. Also, BMAs promote tumour cell oxidative stress [16] and metabolic reprogramming toward a glycolytic phenotype [17]. BMAT is crucial in the development of blood disease, in which BMAs directly interact with blood tumour cells in the bone marrow. In addition, BMAT has been identified as a key driver in the progression of multiple myeloma [1,18,19].

## 3. Adipocytes as Source of Factors in Tumour Stroma: Adiponectin and Leptin

Adipokines are soluble factors with hormone-like functions secreted by adipocytes. The most extensively studied are leptin, adiponectin, inflammatory factors such as interleukin (IL)-6, and tumour necrosis factor (TNF)-α, autotaxin, and hepatocyte growth factor (HGF). In the stroma of breast cancer, adipocytes, along with fibroblasts, macrophages, and cancer cells produce adipokines such as leptin, adiponectin, autotoxin, and IL-6. Breast cancer cells express adipokines’ receptors; adipokines can induce initiation, progression, and metastasis of breast cancer cells with different effects.

Adipokines activate several signalling networks in target cells, including the JAK/STAT, AKT, and ERK1/2 signalling pathways, which are frequently activated in tumour tissues [20,21]. These pathways are associated with cell migration, proliferation, angiogenesis, fibrosis, and apoptosis. Moreover, IL-6 or leptin can increase procollagen-lysine, 2-oxoglutarate 5-dioxygenase 2 (PLOD2) expression in breast cancer cells through the activation of the JAK/STAT3 and the AKT signalling pathway. PLOD2 contributes to the formation of triple helical pro-collagen molecules by triggering the hydroxylation of collagen lysine residues [22,23]. PLOD2 deregulation has been described in sarcoma, breast cancer, glioma, lung cancer, and cervical cancer [23,24,25,26,27].

### 3.1. Adiponectin

Adiponectin (ACRP30), a peptide hormone of 30kDa, plays a major role in glucose metabolism and energy homeostasis [28]. After its secretion by adipocytes as a monomeric protein, adiponectin oligomerizes to form low-molecular-weight, high-molecular-weight, and multimeric complexes [29]. Adiponectin binds to seven pass receptors, AdipoR1 and AdipoR2, and acts centrally to enhance energy homeostasis by increasing energy expenditure through the activation of hypothalamic leptin and insulin signalling pathways. It also improves glucose homeostasis by attenuating insulin resistance. Adiponectin influences adipose tissue functions via autocrine effects: *ADIPOQ* gene overexpression increases the number of adipocytes and adipose tissue mass [18].

WAT was once considered the main source of adiponectin; more recently it was reported that also BMAT secretes a large amount of adiponectin chiefly in the presence of cancer cells [30,31]. Though adiponectin is produced by adipocytes, circulating adiponectin levels in the obese are lower than in the non-obese (the so-called adiponectin paradox) [32] and the increase in serum adiponectin is associated with weight loss (i.e., in patients with anorexia nervosa or in animal models of caloric restriction) [33,34]. Adiponectin is considered a “good” adipokine because of its anti-inflammatory, anti-atherogenic, and insulin-sensitizing properties.

The relationship between adiponectin and bone cells is complex and not completely elucidated. Studies have reported an osteogenic property of adiponectin, as the adipokine is able to stimulate osteogenic differentiation of bone marrow mesenchymal stromal cells and adipose-derived stem cells vs. the osteogenic lineage [35,36].

#### Adiponectin and Breast Cancer/Bone Metastasis

Numerous studies have attempted to shed light on the role of adiponectin in carcinogenesis. 

The in vitro exposure of cancer cell lines (e.g., breast, liver, colon, stomach, and endometrium) to adiponectin inhibits proliferation and induces apoptosis [37,38,39,40,41]. Though it has been reported that adiponectin negatively influences breast carcinogenesis [42], the question remains open and the answer probably depends on hormonal status (ER-PR expression in particular) [43]. Several in vivo and in vitro studies demonstrated that adiponectin in ER negative breast cancer suppresses cell proliferation, invasion, and migration and induces cell growth arrest and apoptosis [37,42,44,45,46], whereas in ER positive breast cancer it increases cell proliferation [47,48]. The review by Panno et al. explains the effect of adiponectin on ER positive and ER negative breast cancer cells [49].

It has also been reported that adiponectin inhibits the metastatic process via suppression of the adhesion, invasion, and migration of breast cancer cells through activation of the AMPK/S6K axis and upregulation of LKB1 [50]. Other studies reported that an increase in the level of globular adiponectin in tumour microenvironment autophagy supports the early stages of metastatic progression [51].

Adiponectin has been found in exosomes derived from adipocytes. Exosomes are extracellular vesicles that mediate cell-to-cell signalling in the tumour microenvironment. Exosomes from human adipose-derived mesenchymal stem cells induce proliferation and migration of breast cancer cells [52] and exosomes secreted by preadipocytes also regulate breast tumour stem cell formation and migration [53]. Further research is needed to discern the role of adiponectin in this context.

### 3.2. Leptin

Leptin, a hormone produced primarily by adipocytes, coordinates energy homeostasis by signalling from adipose tissue to the hypothalamus; its synthesis and plasma concentration increase proportionally to adipose tissue mass. Leptin acts by binding to leptin receptors (Ob-Rs) encoded by the *LEPR* gene and members of the family of class I cytokine receptors. Ob-R presents six isoforms (Ob-Ra, Ob-Rb, Ob-Rc, Ob-Rd, Ob-Re, and Ob-Rf) generated by alternative splicing of the gene. Leptin produces different effects in various organs by binding to central or peripheral receptors. As regards its effects on bone remodelling, central leptin receptors mediate bone loss, whereas the binding of leptin to peripheral receptors results in an increase in bone mass [54,55].

Leptin helps regulate bone health by modulating bone density and growth and adiposity. In the bone marrow, mature adipocytes release leptin, which in turn enhances the formation of BMAs by binding to Ob-R on bone marrow mesenchymal stem cells (BMSCs) to promote their adipogenesis [56]. More importantly, Ob-R (+) BMSCs are the main source of BMAs [57]. Bone cells, including osteoblasts, secrete low amounts of leptin [58,59]. It has been reported that, in the primary culture of human osteoblasts, leptin is expressed and secreted during the late maturation phase to promote bone mineralization [58]. Osteoblasts and adipocytes both originate from mesenchymal stem cells and share common markers.

#### 3.2.1. Leptin and Breast Cancer

Studies using both in vivo and in vitro experimental models have extensively demonstrated the involvement of leptin in many aspects of breast cancer biology starting from the early stages of primary tumour to metastatic progression. In addition to adipose tissue, cancer cells can also secrete leptin and overexpress leptin receptors.

Studies on breast cancer patients have revealed an association between higher serum leptin levels and aggressive malignant tumour features. Leptin expression in normal breast tissue adjacent to ductal carcinoma and its absence in the breast tissue of healthy adults suggests that leptin is involved in the early stages of breast cancer tumorigenesis [60]. Nevertheless, while some studies found a negative correlation between serum leptin levels and breast cancer development in premenopausal women but a positive one in postmenopausal women [61], other studies reported no association between serum leptin and breast cancer development [62].

In their meta-analysis to evaluate the association between serum leptin levels and breast cancer risk [63], Gu et al. reported that the apparent discrepancies among the findings were related to the heterogeneity across the studies: different genetic backgrounds, different stages and types of breast cancer, different analytic methods to measure serum leptin levels, different menstrual and treatment status of breast cancer patients, different demographics and clinical characteristics.

Tumour tissue expression of leptin and/or of Ob-R has been found to correlate with aggressive tumour behaviour [64]. Adipocyte-secreted leptin and IL-6 have a paracrine effect on nearby breast cancer cells. One of the mechanisms through which leptin causes breast cancer progression is the regulation of breast cancer stem cell (CSC) activity by modulating the many signalling pathways (i.e., Notch, Wnt, mTOR, STAT3, HER2/Erb, and IGF pathways) and transcription factors (i.e., HIF-1 and NFκB) [65,66,67,68,69]. Leptin signalling in CSC is also involved in cancer recurrence and drug resistance. 

Guo et al. reported a complex crosstalk between leptin, Notch, and Interleukin-1 (IL-1) (NILCO, Notch, IL-1, and leptin crosstalk outcome) that seems to be the integration of developmental, pro-inflammatory, and pro-angiogenic signals critical for leptin-induced cell proliferation/migration and regulation of VEGF/VEGFR-2 in breast cancer [65]. In obese breast cancer patients with often chronically elevated leptin levels, leptin NILCO signalling seems to provide a link between obesity and cancer [64]. The authors speculated that targeting NILCO may be a new pharmacological strategy to control breast cancer growth and angiogenesis [65,70].

Recently, Giordano C et al. found that leptin regulates exosome biogenesis and release in different models of breast cancer cells via the leptin receptor/Hsp90 axis. This additional mechanism in cell-to-cell communication is part of a biological process that is crucial for key events in breast cancer development and progression [71]. The authors suggested that impairing exosome secretion and interrupting the dangerous cell-to-cell crosstalk could counteract cancer progression. Furthermore, knowledge of the signals in the biogenesis of exosome in breast cancer (i.e., leptin signalling in obese patients) will be important for the design of new therapies [71].

As regards the tumour microenvironment within the mammary gland, leptin can modify the tumour to modulate migration of endothelial cells, angiogenesis, macrophage phenotypes and functions, and recruitment of monocytes and neutrophils. The interaction of leptin with cytokines can also influence breast cancer progression: leptin acts on both tumour cells and tumour stroma to secrete inflammatory cytokines such as IL-1, IL-6, TNF-α, and growth factors.

#### 3.2.2. Leptin and Bone Metastasis

In addition to its effect on breast cancer growth, leptin may also play an important role in bone metastasis. A growing body of evidence shows that leptin may promote epithelial to mesenchymal transition (EMT) by up-regulating the expression of EMT-and metastasis-related genes (Twist2, Foxc2, Vim, Akt3, and Sox2) in the mouse mammary tumour virus (MMTV)-Wnt-1 transgenic mouse model of triple-negative breast cancer [72], highlighting the potential roles of leptin in metastasis development. Bowers and colleagues hypothesized that regulation of the EMT gene, via stimulation of the JAK2/STAT3 and/or PI3K/AKT pathways, may mediate the leptin-induced CSC/EMT-related phenotype.

Recently, Juárez-Cruz et al. hypothesized that leptin promotes FAK and Src activation in breast cancer cells and induces cell migration, metalloproteases secretion (MMP2 and MMP9), and invasion. They suggested that leptin is associated with the establishment of a more aggressive phenotype of cancer cells that leads to local invasion and to the formation of metastases [73].

Leptin is known to influence tumour-associated macrophages (TAMs), a critical component in the tumour microenvironment and a major source of inflammatory cytokines [74]. Stimulation of macrophage function by leptin leads to the release of pro-inflammatory cytokines, resulting in pro-inflammatory effects. Clinical evidence shows that the presence of macrophages within the tumour microenvironment correlates with poor prognosis in cancer patients. TAMs may play a major role in cancer progression, including EMT induction. Analysis of the transcriptome of TAMs in murine models of breast cancer indicated that an enrichment in macrophage transcripts is predictive of poor prognosis and reduced survival [75]. Cao and colleagues showed that leptin, by triggering M2 macrophage-related cytokine IL-18 production, contributes to tumour progression and the development of metastasis. These extremely important results add another link (leptin-Ob-R-IL-18) to the complex interaction between the tumour microenvironment and breast cancer cells that drives cancer progression [76].

The expression of leptin and/or Ob-R by tumour tissue indicates aggressive behaviour. Ishikawa et al. reported that distant metastasis was detected in patients with Ob-R-positive tumours overexpressing leptin, whereas it was absent in patients with tumours that lacked Ob-R expression or leptin overexpression [64].

He et al. found that adipocyte-derived IL-6 and leptin promote PLOD2 expression in breast cancer, an event that promotes breast cancer metastasis [77]. In addition, fat tissue accumulation may contribute to enhance the expression of PLOD2 [69]. The availability of PLOD2 is one way by which IL-6 and leptin may influence collagen re-organization to create a “highway” for cancer cell migration and invasion to promote metastasis [78].

Leptin can affect also the adhesion of cancer cells to the extracellular matrix (ECM) and the proteolysis of ECM components [79,80], essential steps in tumour metastasis. Tsai et al. identified a soluble intercellular adhesion molecule (sICAM)-1 as a downstream effector of leptin: the adhesion molecule is able to induce osteoclastogenesis. With their in vivo study, the authors showed that, in the bone microenvironment, leptin enhanced sICAM-1 production by tumour cells and induced bone erosion. Injection of ICAM-neutralizing antibody reduced the osteolytic process in tumour-bearing mice. These findings led the authors to speculate that sICAM could be a potential therapeutic target or diagnostic tool for bone metastases from lung and breast cancer [81].

## 4. The RNA-Binding Protein Src-Associated in Mitosis of 68 kDa (Sam68)

Src-associated in mitosis of 68 kDa (Sam68) was originally discovered as a protein that is physically associated with and phosphorylated by c-Src during mitosis [82]. Sam68, an RNA-binding protein (a STAR, signal transduction and activation of RNA family member) that links cellular signalling to RNA processing, is markedly expressed in breast cancer cell and tissues. Sam68 has been shown to exert a dual role as an RNA-binding protein and as a docking protein in different cellular contexts [83,84].

Sam68 presents protein–protein interaction motifs that enable it to function as a scaffold protein in multiple signal transduction pathways and in transcription complexes. Moreover, as an RNA-binding protein, Sam68 can modulate alternative splicing and translation of target mRNAs. Sam68 participates in the signal transduction of various receptors, including insulin, T-cell receptor, as well as EGF, HGF/Met, TNFR1, and leptin.

Studies have produced conflicting findings for Sam68 involvement in cancer; the discrepancies arise from the cellular context and the cell type considered. Sam68 was originally defined as a tumour suppressor protein [85,86], however, direct investigation of Sam68 expression and function in cancer cells suggest a pro-oncogenic role for the protein [87,88].

**Figure 3:**
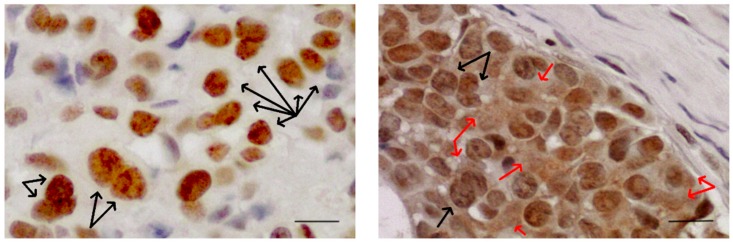
Sam68 is found in distinct sub-nuclear structures SLM/Sam68 nuclear bodies (SNBs, black arrows) enriched in cancer cells. Sam68 in cytoplasm (red arrows) is significantly associated with poor prognosis and progression in different types of tumours. Images of bone metastases from breast cancer. Scale bar= 10μm. Original figure courtesy of the author.

It is now known that Sam68 interacts with the SH3 domain of different members of the Src kinase family, like BRK [89], FYN [90], and Itk/Tec/BTK [91], all involved in different aspects of cell transformation. Sam68 is usually found in the nucleus where it is concentrated in distinct sub-nuclear structures (nuclear speckles) SLM/Sam68 nuclear bodies (SNBs, indicated by black arrows in Figure 3), structures that are enriched in cancer cells [92]. Sam68 in the cytoplasm is significantly associated with poor prognosis and progression in renal cell carcinoma [93], cervical cancer [94], and breast cancer [95].

Moreover, the deregulation of SRC and AKT pathways may be involved in the oncogenic function of Sam68 in the cytoplasm. The subcellular localization of Sam68 (cytoplasm or nucleus) reflects its different functions in various signalling pathways. It can contribute to neoplastic transformation or tumour progression through different molecular mechanisms in different cancer types or cellular contexts [96]. Sam68 in the cytoplasm in advanced tumour stages may contribute to neoplastic transformation through the translational enhancement of specific mRNAs [97].

The upregulation of Sam68 is reported in prostate cancer and in highly proliferative lesions of human breast cancer. Its downregulation seems to promote inhibition of cell proliferation and sensitization of cells to apoptosis induced by chemotherapeutic agents in prostate cancer [98], while silencing of Sam68 in breast cancer cells inhibits cancer proliferation. In their study on Sam68-null mice, Richard et al. found that the absence of Sam68 delays mammary tumorigenesis, metastasis, and cell migration, giving further evidence for the role of Sam68 in cell transformation [88]. Moreover, they demonstrated that activated Src and FAK kinases are present in Sam68-null mice but not wild type mice, suggesting that Sam68 may be a modulator of these pathways during cancer progression [88].

Sam68 is regulated at the post-translational level by phosphorylation [99], acetylation [100], methylation [101], and SUMO (small ubiquitin-like modifier) modification (SUMOylation, a post-translational modification analogous to ubiquitylation and involving the addition of SUMOs to proteins instead of ubiquitin) [102]. Such modifications can affect its subcellular localization, interaction with signalling proteins, as well as affinity for target RNAs [103,104]. Tyrosine phosphorylation regulates both the signal transduction and RNA-binding properties of Sam68 [89,105]. Tyrosine phosphorylation of Sam68 is elevated in human breast and prostate tumour tissues and cell lines [100,104,105,106]. Sam68 expression level and phosphorylation status play a role in tumorigenesis and indicate Sam68 as a proto-oncogene [107].

In human breast cancer cells, Sam68 participates in leptin receptor signalling by modulating the trophic effects of the hormone in cellular proliferation and growth. Leptin and insulin increase Sam68 expression in breast cancer cell lines, with an increase in its tyrosine phosphorylation [97]. Pérez-Pérez et al. demonstrated that Sam68 plays a role in leptin and insulin signal transduction pathways in three different breast carcinoma cell lines by activating MAPK and PI3K signalling pathways and partially modulating the expression of IRS1 [108]. Sam68 may be considered a point of convergence for various different signals involved in breast cancer growth and progression. Finally, Sam68 intervenes in EMT/Mesenchymal to epithelial transition (MET) programs. Because of the relevance of EMT/MET in the formation of metastases, Sam68 may play a role in cancer progression. 

## 5. Alternative Splicing Regulates EMT: Role of Sam68

Alternative splicing of pre-mRNA is a major driver of protein diversity and affects more than 95% of multi-exonic coding human genes [109]. Upregulation of endogenous alternatively spliced tumorigenic variants is frequently observed in many cancers such as the pro-angiogenic splice variant of vascular endothelial growth factor (VEGF_165_), an EMT-inducing isoform of Rac1 (Rac1b), and the cancer-initiating form of protein kinase C (PKC) betaII [110,111,112,113].

Alternative splicing generates multiple mRNAs from a single transcript. It is fundamental for proteomic diversity and control of gene expression. In addition, it is involved in essential biological events such as development, cell differentiation, organogenesis, and response to environmental stimuli. The deregulation of alternative splicing leads to tumour biogenesis and progression [114,115].

Neoplastic cells positively exploit the biological flexibility that, via the production of splice variant proteins, promotes tumour growth and survival. Proteome remodelling by alternative splicing enables cancer cells to produce proteins that meet the needs of the tumour as it grows and spreads.

Many cancer-associated genes are regulated via alternative splicing, suggesting a significant role for this post-transcriptional regulatory mechanism in the production of oncogenes and tumour suppressors. Aberrant regulation of alternative splicing is emerging as a basic step in oncogenesis [116] and it is considered another hallmark feature of cancer among the growing list of tumour growth characteristics [117].

A critical step in cancer cell dissemination and progression is the transition towards a mesenchymal phenotype via the activation of EMT. EMT requires robust reprogramming of gene expression. Mounting evidence indicates an important contribution of alternative splicing regulation in EMT; the chief factors involved in EMT are regulated post-transcriptionally by alternative splicing. Tissue-specific alternative splicing regulators include the neuron specific RNA-binding proteins 1/2 (NOVA1/2), the polypyrimidine tract binding protein 2 (PTBP2), the serine/arginine repetitive matrix 4 protein (SRRM4), and members of the RBFOX, Muscle blind-like (MBNL) proteins, CUGBP Elav-like family (CELF), T-cell intracellular antigen (TIA), epithelial splicing regulatory protein (ESRP) and STAR families. Sam68 was the first “hub factor” to be identified that translates extracellular stimuli to pre-mRNA processing of specific target genes in the nucleus and causes specific RNA splicing decisions [118]. The *CD44* gene is an example in which Sam68 exerts a link between signal transduction cascades and alternative splicing [119]. The alternative splicing generated CD44v, epithelial isoforms, or CD44s, mesenchymal isoform; the transition from CD44v to CD44s is thought to play a central role in EMT. Other Sam68/EMT/alternative splicing interactions were described by Valacca and colleagues, who reported that Sam68 might contribute to the malignant transformation of epithelial cancers by inducing the proto-oncogene *SRSF1* (serine/arginine-rich splicing factor 1, historically known as SF2/ASF) and stimulating a post-transcriptionally regulated EMT [107]. Also, in mesenchymal cells Sam68 phosphorylation by the extracellular regulated kinase 1/2 (ERK1/2) increases the full-length transcript of SRSF1 (splicing factor), thus increasing SRSF1 protein levels that, in turn, drive a ΔRon (an oncogenic variant of constitutively active Ron) that triggers EMT [120]. Diffusible factors secreted by epithelial cells (human colon carcinoma-derived SW480 cells grown at high density) repress ERK1/2 activity (in SW480 cells grown at low density) and Sam68 phosphorylation, thus reducing SRSF1 protein levels and promoting the inclusion of Ron exon 11 and MET [107]. Anti-metastatic therapeutic strategies may be addressed to cancer-specific splicing variants involved in EMT [109].

Moreover, Sam68 regulates the apoptotic gene *BCL-X* via alternative splicing in cancer cells [105], leading to an alteration in the BCL-XS/BCL-XL ratio (pro-apoptotic and anti-apoptotic, respectively) [105]. Tyrosine phosphorylation of Sam68 may be one way that cancer cells protect against apoptosis. 

## 6. Conclusions

BMAT appears to play a pivotal role in creating a microenvironment suitable for the colonization of bone by cancer cells via the release of numerous different factors. In addition, obesity mediates its effect on cancer progression via the dysregulation of adipokine release. Table 1 compares the role of the three molecules in the onset and progression of breast cancer. Leptin and adiponectin may exert antagonistic molecular effects on cancer progression. Further research into this aspect is particularly important because published data are sometimes conflicting. Leptin and adiponectin released into the tumor microenvironment at different times have opposite effects on tumor growth. Intracellular signals could favor the effect of one factor over the other. Indeed, leptin can intervene in different ways to sustain metastatic cells. The parallelism between leptin and Sam68 in their ability to promote cancer cells indicates that Sam68 is an intracellular mediator involved in leptin activity in cancer cells. The leptin-Sam68 axis appears to be essential for the growth and progression of breast cancer. Some observations may be relevant only for a particular cellular context. In vitro studies evaluating the effect of a microenvironment factor on tumoral cell lines do not take into account the simultaneous presence of other molecules, as occurs in vivo, that interact with each other. Advances in this field may open new insights into therapeutic opportunities for treating bone metastasis.

## Figures and Tables

**Table 1:** The role of leptin, adiponectin, and Sam68 in breast cancer: (+) positive regulation (activation), (-) negative regulation (inhibition), (+/-) positive or negative regulation depending on phosphorylation status.

Breast Cancer Progress	Leptin	Adiponectin	Sam68
Tumourigenesis	+ [60,121,122]	− [123,124,125]	+ [94,125]
Tumour progression	+ [126,127,128]	− [42,50,129] or + [51,130]	+ [96,125]
Cancer cell invasion	+ [131,132]	− [50,74] or + [130,133]	+ [134]
Metastasis	+ [72,135]	− [50] or + [51]	+ [134]
Angiogenesis	+ [65,136,137]	− [138] or + [139,140]	?
Tumour cell apoptosis	− [127,141]	− [38] or + [138]	+ [112,142]
Cancer stem cells (CSC)	+ [69,72,135]	− [143]	+ [144]
EMT	+ [145,146,147,148]	? (in breast cancer) − (in lung and prostate carcinoma [149,150])	+/− [107,151]

## Abbreviations

MSC	Mesenchymal stem cell
STAR	Signal Transduction and Activation of RNA
WAT	White adipose tissue
BAT	Brown adipose tissue
BMAT	Bone marrow adipose tissue
cBMA	Constitutive bone marrow adipocytes
rBMA	Regulated bone marrow adipocytes
PLOD2	2-oxoglutarate 5-dioxygenase 2
AMPK	Adenosine-monophosphate activated protein kinase
LKB1	Liver kinase B1
Ob-R	Leptin receptor
BMSC	Bone marrow mesenchymal stem cell
CSC	Cancer stem cell
NILCO	Notch, Interleukin-1, and leptin crosstalk outcome
EMT	Epithelial to mesenchymal transition
TAMs	Tumor-associate macrophages
ECM	Extracellular matrix
sICAM	Soluble intercellular adhesion molecules
Sam68	Src-associated in mitosis 68
MET	Mesenchymal to epithelial transition
SRSF1	Serine/arginine-rich splicing factor 1
